# Short-term outcomes of unfractionated heparin vs. low molecular weight heparin in trans-radial coronary angiography: a comparative study

**DOI:** 10.3389/fcvm.2025.1535463

**Published:** 2025-10-09

**Authors:** Salma Taha, Mahmoud Ibrahim Mohamed Ibrahim Sakr, Mohamed Salah El Deen Abd El Salaam, Mohamed Mahmoud Ahmed, Mohamed Aboel-Kassem F. Abdelmegid

**Affiliations:** ^1^ Cardiovascular Medicine Department, Faculty of Medicine, Assiut University, Assiut, Egypt; ^2^ Department of Cardiology, Sohag Heart and Liver Institute, Sohag, Egypt; ^3^ Department of Internal Medicine, Faculty of Physiotherapy, Sphinx University, Assiut, Egypt; ^4^ Department of Basic Medical Sciences, Badr University, Assiut, Egypt; ^5^ Assiut Branch-Alazhar University, Assiut, Egypt

**Keywords:** trans-radial coronary angiography, radial artery occlusion (RAO), low molecular weight heparin (LMWH), unfractionated heparin (UFH), percutaneous coronary intervention (PCI)

## Abstract

**Background:**

Trans-radial access (TRA) for coronary angiography is preferred for its lower complication rates than trans-femoral access. However, radial artery occlusion (RAO) remains a significant concern.

**Objective:**

The objective of this study was to assess the efficacy of low molecular weight heparin (LMWH) compared to unfractionated heparin (UFH) in preventing radial artery occlusion (RAO) and associated problems during trans-radial access (TRA) for coronary angiography.

**Methods:**

In this moderate sized clinical prospective study conducted from March 2019 to January 2020 at Al Azhar Assiut University Hospital and Sohag Cardiac and GIT Center, 400 patients undergoing elective coronary angiography via TRA were divided into two groups. Group A (*n* = 200) received UFH, and Group B (*n* = 200) received LMWH (enoxaparin). Parameters assessed included radial artery diameter, flow volume, intima-media thickness (IMT), and complications post-procedure.

**Results:**

Except for gender distribution, no significant differences at baseline were identified between the groups. Although both groups showed slight changes in arterial diameter and flow volume with a small rise in IMT after 10 days, there were notable differences in radial artery occlusion rates. The UFH group had an occlusion rate of 3.5%, while the LMWH group had a rate of 0.5% (*P* = 0.032). The groups exhibited no significant difference in other consequences, such as hematoma and arteriovenous fistula.

**Conclusions:**

LMWH (enoxaparin) administration via the radial sheath during TRA is a safe and effective strategy for reducing the incidence of RAO compared with UFH without increasing other procedural complications. This suggests that LMWH may be preferred for patients undergoing TRA for coronary angiography to minimize the risk of RAO.

## Introduction

Coronary angiography is the gold standard for diagnosing and implementing atherosclerotic coronary artery disease treatment techniques. Coronary angiography can be conducted using the femoral, radial, brachial, ulnar, or axillary arteries (1).

Historically, TFA was the preferred route. However, vascular access site complications such as bleeding, hematoma, arteriovenous fistula, or pseudo-aneurysm are quite common after TFA procedures. Trans-radial access (TRA) has been increasingly used because of the lower rate of access site complications, shorter hospital stays, improved patient comfort, and safer hemostasis than trans-femoral access (2, 3).

Radial artery occlusion (RAO) is a quiescent consequence of TRA that rarely leads to acute hand ischemia necessitating surgery since the hand has a dual vascular supply from the palmar arch. RAO is often overlooked; over 50% of operators do not assess radial artery patency before discharge (4). Several studies have reported higher RAO of 32.9%. Thrombosis has been claimed as the cause, and retrieval of occluding thrombotic plugs from radial artery has been demonstrated (5).

Radial artery occlusion rate had been demonstrated to be as high as 70% when heparin was not used (6). Currently, U.H. is used universally in trans-radial procedures, reducing the RAO rate to 5%–10%. The role and efficacy of other anticoagulants during TRA is unclear. Although low molecular weight heparins (LMWH) have several advantages over U.H. enoxaparin, they have been approved for anticoagulation during coronary interventions (7, 8).

Enoxaparin is being used increasingly as the anticoagulant of choice in cardiology. Its effectiveness has been proven in patients with acute coronary syndromes undergoing interventional procedures. Enoxaparin provides a more consistent and predictable degree of anticoagulation than UFH, eliminating the need to monitor the anticoagulation level (9).

Our study aims to assess the efficiency of low molecular weight heparin (LMWH) and unfractionated heparin trans-radial access with a primary endpoint to assess radial artery occlusion rate and a secondary endpoint to assess other complications.

## Patients and methods

### Patients

**Figure 1:**
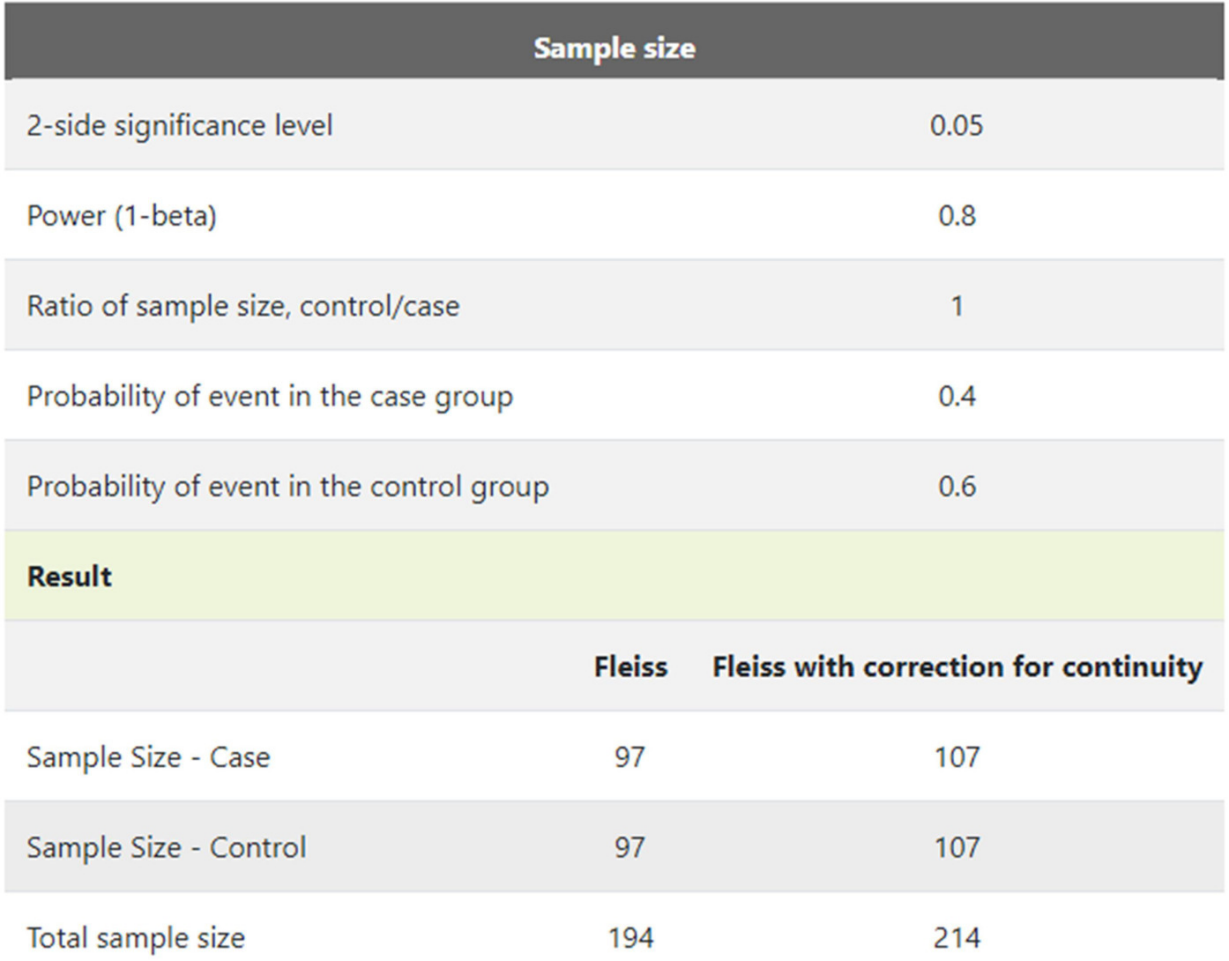
Sample size calculation.

This moderate sized clinical prospective randomised study included 400 patients (Figure 1) admitted for elective coronary angiography from March 2019 to January 2020 in the coronary cath unit, department of Cardiology Al-Azhar University Hospital and Sohag Cardiac and GIT Center. Patients were divided into two groups: group A included 200 patients who received heparin sulfate (70–100 IU/kg) via the radial artery through the radial sheath, and Group B included 200 patients who received a total dose of low-molecular-weight heparin; enoxaparin 1 mg/kg injected via the radial artery through the radial sheath. The study was conducted with the utmost ethical considerations; all patients provided informed consent, and the ethical committee approved the study for research involving human beings, along with administrative permission.

**Inclusion criteria:** All patients scheduled for an interventional or diagnostic cardiac procedure deemed suitable for trans-radial access met the inclusion criteria.

**Exclusion criteria were** the absence of radial artery pulsations, failed previous radial access, chronic renal failure, expected severe hemodynamic deterioration, older age >75 years, contraindications to unfractionated heparin or enoxaparin, treatment with injectable anticoagulants for less than 8 h ago, patients suspected of CABG, and patients need shunt for renal dialysis.

### Methods

All patients were subjected to the following:

1. Complete History and Physical examinations.
2. 12 Lead ECG using the Fukoda FX 7102 ECG
3. Echocardiogram using devices of semiens sonoline G60S and G.E. Vivid E95.
4. Laboratory assessment of serum creatinine, hepatitis markers, and coagulation profile.
5. Preprocedural measures.

Palpation and the Doppler (main tool of outcome) assessments of the radial artery were performed by the same operator before the procedure and at discharge and 10 days after the procedure for assessment of radial artery patency, using a commercially available instrument (Aloka pro sound 4000, semiens sonoline G60S and G.E. Vivid E95) with harmonic MHz variable frequency phased-array transducers. Radial artery diameter, intima-media thickness, and flow velocity were measured before, at discharge, and 10 days following procedure by the same operator.

Patency of the radial artery was more accurately assessed through clinical testing, such as the reverse Barbeau's test and Doppler ultrasonography. The ulnar artery was occluded in the former method, and a pulse oximeter was located on the ipsilateral thumb. The absence of plethysmographic waveforms was a sign of RAO. The latter method utilized color Doppler ultrasound to evaluate blood flow and provide structural imaging of the arteries. Occlusion was indicated by the absence of flow in the radial artery. The laser Doppler scan was used as a novel, noninvasive method that had the potential to facilitate the rapid diagnosis of RAO.

### Procedure details

#### Transradial catheterization procedure

They are performed under sterile conditions starting with local anesthesia at the puncture site. Followed by radial artery access using a 20-gauge needle and a 0.025-inch wire through a 6-French sheath. Nitro-glycerine (200 μg) was administered through the sheath for vessel dilation. Patients received either heparin sulfate or low-molecular-weight heparin (enoxaparin), depending on the treatment group. The procedure concluded with angiography performed with 6-French coronary catheters.

#### Haemostasis procedures

Following angiography, all sheaths were removed, and a radial compression device (T.R. band) was placed around the wrist, inflated with 15 ml of air to achieve hemostasis. The inflation pressure of the T.R. band was gradually decreased at 15, 30, and 60 min by removing 3–5 ml of air. A light dressing was applied to the site following removal of the compression device.

#### Endpoints and definitions

**Figure 2:**
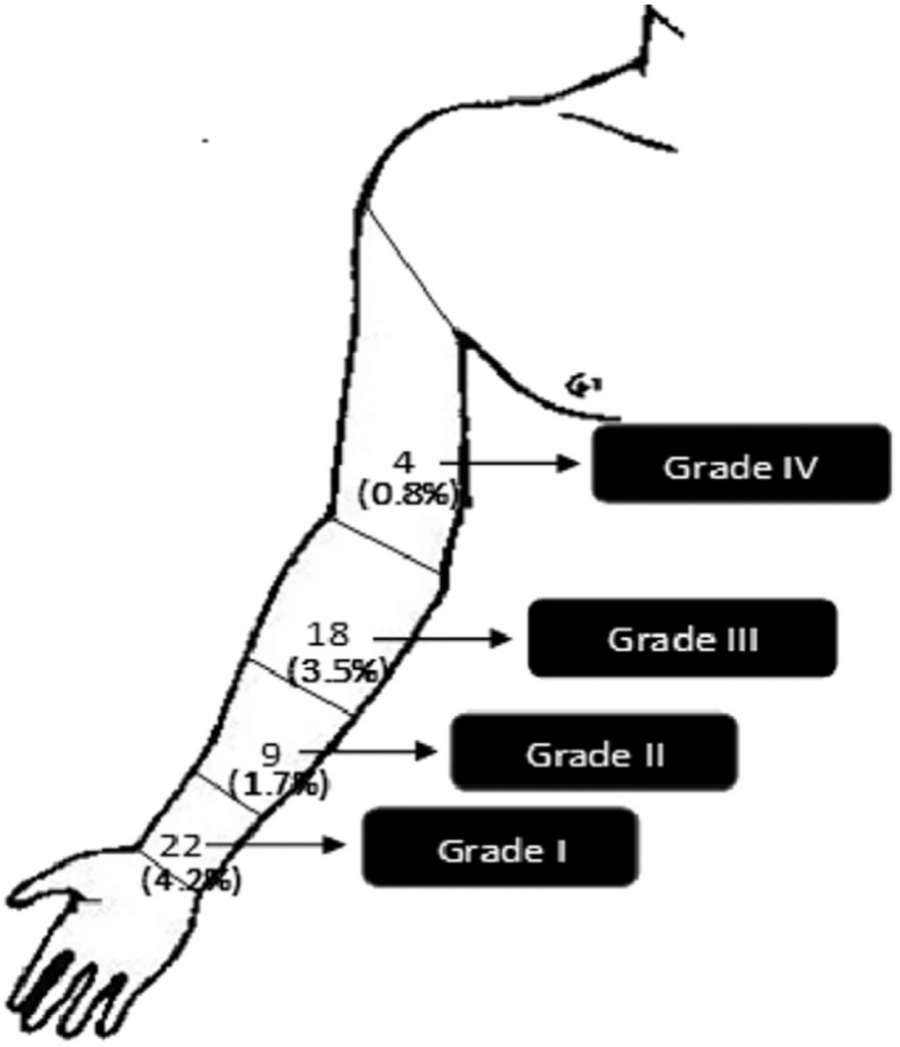
EASY (early discharge after transradial stenting of coronary arteries study) hematoma grading.

The study aimed to measure the incidence of radial artery occlusion (RAO) post-procedure such as secondary objectives included grading forearm hematomas using EASY grading (10), Figure 2 from grade I (<5 cm) to grade V according to their size (compartment syndrome) (11) and examining other local complications like bleeding, pseudo-aneurysm, and arteriovenous fistula.

#### Ethical considerations

The study was done after being accepted by the Research Ethics Committee, Al-Azhar University (Approval code: MS-16-9-2022). Prior to enrollment, patients provided written informed consent. The consent form explicitly outlined their agreement to participate in the study and for the publication of data, ensuring that their confidentiality and privacy would be protected. This work has been carried out per The Code of Ethics of the World Medical Association (Declaration of Helsinki) for studies involving humans. Both the patients and the operators were blinded to the research study.

### Statistical analysis

Statistical analysis: Data was analyzed using STATA intercooled version 12.1. Quantitative data was represented as mean, standard deviation, median, and range. Data was analyzed using a student *t*-test to compare the means of the two groups. When the data were not normally distributed, the Mann–Whitney test was used. Qualitative data was presented as numbers and percentages and compared using the Chi-square or Fisher exact tests. The *P*-value will be considered significant if it is less than 0.05.

## Results

### Demographic data, laboratory data, and Doppler data in both groups

The baseline characteristics had no significant differences between the two groups except for gender, as there was a predominance of males in both groups. Laboratory assessment before catheterization showed no significant differences. Significant differences were found between the radial artery diameter, flow volume, and intima-media thickness (IMT) groups. The heparin group had a minor change in arterial diameter and flow volume, with a slight increase in IMT over 10 days. The enoxaparin group showed similar trends but started with different baseline values. Despite initial differences, overall radial artery diameter remained consistent across all participants, indicating distinct impacts of each anticoagulant on vascular parameters post-procedure (Table 1).

**Table 1:** Demographic data, laboratory data, and Doppler data in both groups.

Variables	UFH	LMWH	Total (*N*/%) 400	*P*-value
Male	151 (75.5)	127 (63.5)	278 (69.5)	**0.005** *
Age (years)	58.7 ± 8.4	57.9 ± 10.2	58.3 ± 9.3	0.38
Weight (Kg)	82.2 ± 11.1	83.9 ± 14.3	83.1 ± 12.8	0.19
Height (cm)	168.2 ± 5.8	168.3 ± 7.4	186.2 ± 6.6	0.85
BMI (Kg/m^2^)	29.1 ± 3.5	29.7 ± 5.2	29.4 ± 4.4	0.13
DM (*N*/%)	119 (60)	96 (48)	216 (53.9)	0.014
HTN (mmHg)	136 (68)	113 (56.5)	249 (62.3)	**0.02** *
Smoking (*N*/%)	124 (62)	95 (47.5)	219 (55)	**0.003** *
INR	1.1 ± 0.1	1.1 ± 0.1	1.1 ± 0.1	0.92
Hepatitis C	29 (14.5)	22 (11)	51 (12.7)	0.40
Hepatitis B	2 (1%)	1 (0.5%)	3 (0.75)	0.40
RA. Diameter before (mm)	2.64 ± 0.23	2.50 ± 0.31	2.62 ± 0.28	**0.000** *
R.A. Diameter after (mm)	2.62 ± 0.23	2.53 ± 0.30	2.60 ± 0.26	**0.000** *
R.A. Diameter 10 days (mm)	2.65 ± 0.21	2.51 ± 0.31	2.61 ± 0.24	**0.000** *
Flow Volume before (cm/s)	13.8 ± 2.5	15 ± 3.2	14.4 ± 3.0	**0.000** *
Flow Volume after (cm/s)	13.2 ± 3.6	14.5 ± 3.6	13.9 ± 3.7	**0.000** *
Flow Volume 10 days (cm/s)	13.2 ± 3.6	14.5 ± 3.6	13.9 ± 3.7	**0.000** *
IMT. Before (mm)	0.71 ± 0.11	0.68 ± 0.11	0.69 ± 0.11	**0.003** *
IMT. After (mm)	0.72 ± 0.11	0.71 ± 0.17	0.71 ± 0.11	0.431
IMT. 10 days (mm)	0.72 ± 0.13	0.71 ± 0.16	0.71 ± 0.10	0.421

IBold values show statistical significance. MT, intima-media thickness; R.A., radial artery; UFH, unfractionated heparin; LMWH, low molecular weight heparin.

*Significant *P*-value.

### Procedural data between heparin and enoxaparin group

Significant differences were found in the used catheter number and fluoroscopy time but not in the number of punctures or procedure time. Specifically, the heparin group (Group A) used fewer catheters (2.3 ± 0.5) compared to the enoxaparin group (Group B), which used 2.5 ± 0.6, with this difference being statistically significant (*P* = 0.01). Both groups had similar mean numbers of punctures and procedure times, indicating no significant difference in these aspects. However, the fluoroscopy time was significantly shorter in the enoxaparin group (3.8 ± 2.5 min) compared to the heparin group (4.7 ± 3.8 min, *P* = 0.005), and a slight but statistically significant difference was observed in compression time, with enoxaparin requiring slightly more time (3.2 ± 0.4 h) compared to heparin (3.1 ± 0.4 h, *P* = 0.002) (Table 2).

**Table 2:** Procedural data between heparin and enoxaparin group.

Variables	UFH	LMWH	Total (*N*/%) 400	*P*-value
Catheters number	2.3 ± 0.5	2.5 ± 0.6	2.4 ± 0.6	**0**.01*
Punctures number	1.3 ± 0.5	1.2 ± 0.4	1.2 ± 0.5	0.06
Procedure time (min)	12.6 ± 6.4	13.6 ± 8.3	13.2 ± 7.4	0.20
Fluoroscopy time (min)	4.7 ± 3.8	3.8 ± 2.5	4.3 ± 3.2	**0**.005*
Compression time (h)	3.1 ± 0.4	3.2 ± 0.4	3.1 ± 0.4	**0**.002*

Bold values show statistical significance. UFH, unfractionated heparin; LMWH, low molecular weight heparin.

*Significant *P*-value.

### Study endpoints or complications of the two groups

A significant difference was found in radial artery occlusion (RAO) rates, with 3.5% in the heparin group vs. 0.5% in the enoxaparin group (*P*-value 0.032). No statistically significant difference was observed between the groups in absent radial pulse rates or the occurrence of mild access site hematoma and arteriovenous (A.V.) fistulas. The study highlighted a specific case of RAO with preserved pulse in the heparin group and reported no cases of bleeding across all participants (Table 3).

**Table 3:** Study endpoints or complications of the two groups:.

Variables	UFH	LMWH	Total (*N*/%) 400	*P*-value
RAO after 24 h	7 (3.5)	1 (0.5)	8 (2)	**0**.032*
RAO 10 days follow up	7 (3.5)	1 (0.5)	8 (2)	**0**.032*
Absent Radial Pulse	6 (3)	1 (0.5)	7 (1.8)	0.06
Hematoma (mild)	13 (6.5)	12 (6)	25 (6.2)	0.83
AV Fistula	1 (0.5)	2 (1)	3 (0.75)	0.56
Bleeding	0	0	0	—

Bold values show statistical significance. RAO, radial artery occlusion; UFH, unfractionated heparin; LMWH, low molecular weight heparin.

*Significant *P*-value.

## Discussion

TRA is more challenging technically, requires more time to master, and can cause radial artery spasms and occlusion, commonly in elderly patients and women (4). Anticoagulation during TRA procedures is mandatory to significantly reduce RAO incidence (4). The present study evaluated the effectiveness of enoxaparin in comparison with UFH during elective diagnostic and/or interventional cardiac procedures (with planning coronary intervention at the same session if needed) administered through the radial sheath to prevent RAO.

The main findings of the present study were that enoxaparin (1 mg per kg) administered through the arterial sheath is effective in preventing RAO. The trans-radial cardiovascular procedures that were documented by Doppler examination in this study resulted in a numerically and statistically significant difference compared to UFH administration. The RAO rate with enoxaparin was (0.5%), and no bleeding or other significant access site complication was observed. In 2007, Pancholy proposed that the primary mechanism of R.A. occlusion is thrombus formation at the puncture site rather than dissection or spasm. The proposal was substantiated by the efficacy of anticoagulants in preventing RAO (12). Additionally, in 2017, Avdikos et al. demonstrated that imaging studies, such as vascular ultrasound, angiography, optical coherence tomography, and histopathological examination of materials aspirated after mechanical recanalization of occluded radial arteries further supported the thrombus formation at puncture site theory (13).

Navarese et al. as meta-analysis, and specifically on STEMI patients demonstrated that many trials prove the clinical performance of I.V. LMWH is better than that of UFH in cardiovascular interventions (14). The use of UFH during PCI is limited by its unpredictable effect, the need for close monitoring, and the uncertainty around optimal levels of activated clotting time; these indicate the need for a better and safer anticoagulation regimen (15).

The RAO rate was demonstrated to be as high as 70% when heparin was not used (16). Currently, UFH is used universally in trans-radial procedures and reduces the RAO rate to 5%–10% (17), consistent with our study, in which the RAO rate with UFH reached 3.5%. According to Duschek et al. (18), anticoagulation is a fundamental therapeutic approach in endovascular interventions. UFH has been the anticoagulant of choice for many years but has been primarily replaced by LMWH because of its biological and pharmaco-kinetic drawbacks. Furthermore, many trials proved that I.V. LMWH performed better clinically than UFH in cardiovascular interventions (18).

Enoxaparin is being used increasingly as the anticoagulant of choice in cardiology. Its effectiveness has been proven in patients with acute coronary syndromes undergoing interventional procedures. Compared to UFH, enoxaparin produces a more consistent and predictable anticoagulation level, eliminating the need to monitor it (19).

In 2010, Feray et al. reported that pooled data analysis from numerous studies suggested that enoxaparin is as practical, or perhaps even slightly more successful than U.H. in patients undergoing elective PCI. Furthermore, they determined that the risk of thrombocytopenia was also lower with enoxaparin (6). In 2012, Silvain et al. stated that in a meta-analysis enoxaparin was superior to unfractionated heparin reducing mortality and hemorrhage outcomes during PCI. This superiority was particularly evident in patients with S.T. elevation myocardial infarction undergoing primary PCI (20). Enoxaparin is the leading low molecular weight heparin with the most significant volume of published information on use in the PCI setting. Enoxaparin provides consistent anticoagulaton without the need for additional intraoperative ACT/PTT monitoring (20).

These studies were consistent with our study, which showed that enoxaparin is superior to UFH with numerically and statistically significant differences in RAO rate, without any observed bleeding or other significant access site complications (20). Anticoagulation with enoxaparin is associated with less platelet reactivity (21). Accordingly, during endovascular interventions, enoxaparin may contribute to a lesser risk of platelet aggregation and thromboembolic events. Enoxaparin significantly reduces major ischemic events compared with UFH in treating thromboembolic diseases (18).

In 2003, Wakeyama et al. mentioned that the significant etiology of intima-media thickening in the radial artery involves using a sheath larger than the radial artery. They stated that the radial artery was indeed injured during the insertion of the sheath. There were no differences in the IMT of the proximal radial artery between repeat-TRI and first-TRI patients, probably because the mean diameter of the proximal radial artery was >2.52 mm, which was consistent with our study showing no significant difference in IMT before and after TRI using 6f sheath (22).

An iatrogenic arteriovenous fistula is a rare vascular complication of trans-radial artery coronary angiography. During access, needle deviation through a venous tributary may result in an undetected combined artery and vein puncture. Due to the benign natural history and presumed spontaneous resolution of arteriovenous fistula, a conservative approach is advised as the initial treatment option, in contrast to surgical ligation (22, 23).

RAO is often overlooked; over 50% of operators do not assess radial artery patency before discharge (24). RAO is a quiescent consequence of TRA that rarely leads to acute hand ischemia necessitating intervention because the hand has a dual vascular supply from the palmar arch (4).

Similarly, in 2018, Kiani et al. demonstrated that the secondary endpoints were not significantly different between the two groups and that the more extensive hematoma was not observed in the UFH group. Nevertheless, one patient in the enoxaparin group was observed to have a medium and one patient to have a large hematoma. These hematomas were successfully treated with intermittent long-standing compression with the cuff of the blood pressure cuff (25).

Our study was limited and weak because it was a single-center study with a small sample size without conducting subgroup analysis, we have carried out a decent clinical study. However, the study could have been considerably strengthened by larger sample size.

The strength of the study is being the first to look at primary prevention of RAO occlusion with LMWH when compared to UFH.

## Conclusions

The advantages of TRA include a reduced occurrence of bleeding and access site complications, enhanced patient satisfaction, and potentially lower overall expenses than transfemoral access. The most significant complication of TRA is RAO, which may serve as a deterrent to numerous operators. The primary cause of RAO is the development of thrombus at the puncture site, rather than dissection or spasm. Enoxaparin administration via the radial sheath during TRA is safe and effective for preventing RAO 24 h to 10 days following the surgery, compared to UFH. Still further studies are needed to confirm the role of LMWH usage in radial artery intervention.

## Data Availability

The original contributions presented in the study are included in the article/Supplementary Material, further inquiries can be directed to the corresponding author.
